# Confidence signalling aids deception in strategic interactions

**DOI:** 10.1038/s41598-025-00279-w

**Published:** 2025-05-02

**Authors:** Briony D. Pulford, Marta Mangiarulo, Andrew M. Colman

**Affiliations:** School of Psychology and Vision Sciences, University of Leicester, Leicester, UK

**Keywords:** Asymmetric information, Confidence heuristic, Deadlock game, Deception, Incomplete information, Prosociality, Psychology, Human behaviour

## Abstract

When two people are motivated solely to coordinate their actions, but one is better informed than the other about how best to achieve this, confidence signalling can facilitate mutually rewarding choices, and the use of this so-called confidence heuristic has been confirmed in experiments using coordination games. To investigate whether confidence signalling can also be used deceptively, we investigated behaviour in strategic games in which the better-informed player can benefit selfishly by misrepresenting confidence signals deliberately. We manipulated the relative quality of information provided to members of 55 dyads who discussed, under incomplete and asymmetric information, a series of problems in which they had to decide which of two shapes was closest in size to a target shape. Monetary incentives were structured according to the Deadlock game. We found that players with superior information felt greater confidence and attempted on a substantial minority of trials to deceive the other player, mainly by withholding the correct answer at the start of the discussion. We conclude that confidence signalling, even without lying, is sometimes used to deceive.

## Introduction

Do people use deceptive signalling in strategic interactions with others whose interests conflict with theirs? If so, what deceptive signals are used, and how do they work? The research discussed in this article addresses these questions experimentally, but to understand the problems in context, it is first necessary to discuss interactions without conflicting interests.

Circumstances frequently arise in everyday social, economic, and political interactions in which people try to work together to achieve a shared goal but have different levels of relevant knowledge about how best to achieve it. For example, a couple may be planning a holiday together, and one of them may know more than the other about the tourist destinations under consideration. In such circumstances (*incomplete* and *asymmetric information* in the terminology of game theory), assuming that the people involved have the opportunity to discuss the problem with each other, they are likely to agree on a better joint decision if they agree to go along with the opinion of the well-informed individual.

Although everyday strategic interactions are typically games of incomplete information, for many decades, most game theorists assumed that incomplete-information games could never be formalized mathematically. Harsanyi (1967)^1^ was the first to show how to do it, with what he called *Bayesian games*. Research in experimental games continues to focus almost exclusively on games of complete information, although some notable studies using games of incomplete information have been reported^2–7^.

Thomas and McFadyen^8^ (1995) developed a formal theory of the role of confidence signalling in such dyadic strategic interactions between people who have shared preferences and are motivated solely to coordinate their actions to achieve an outcome that both prefer. The problem of coordination is trivial if the players can communicate freely with each other before making their decisions and have full knowledge of the payoffs associated with the possible outcomes. Thomas and McFadyen therefore focused on circumstances in which the players are free to communicate but have incomplete and asymmetric information, one player being better-informed than the other. Their analysis rested on two assumptions: first, that when people communicate their beliefs, they tend to signal confidence in proportion to their degree of confidence or certainty, based on their relevant knowledge; and second, that people tend to judge the persuasiveness of a communicated belief according to the confidence with which it is expressed. Thomas and McFadyen formalized these assumptions in a mathematical model that they then used to prove that the *confidence heuristic*, as they called it, reliably implements optimal negotiated solutions in coordination games with asymmetric information.

The confidence heuristic was put forward as a purely theoretical concept. Many years later, Pulford et al.^9^ reported the results of three experiments, using two-player coordination games with incomplete and asymmetric information, that provided strong evidence for the confidence heuristic in practice and showed how it works. These experiments confirmed the basic assumptions that the players with strong evidence tend to be both more confident and more persuasive than their partners with weak evidence. They found that the confidence heuristic worked equally well whether the players discussed the problems face-to-face or through text messaging, suggesting somewhat surprisingly that verbal rather than nonverbal communication is used to signal confidence. The only potential confidence signal observed in their experiments that turned out to have a significant effect was a particular type of response latency: in a typical discussion between the players, the confident player tended to suggest an answer before the other player had made a suggestion. A similar response latency effect had previously been reported by Kimble and Seidel^10^ in an investigation of expression of confidence in answers to trivia questions: people answer quicker (and louder) when they are more confident of the right answer, even in the absence of an audience. Pulford et al.’s findings suggest that the mechanism through which the confidence heuristic works is as follows. The player with strong evidence usually feels significantly more confident than the player with weak evidence; when the players discuss the problem, the better informed and more confident player typically makes the first suggestion about what to choose, and the less informed and less confident player tends to go along with the suggestion of the better-informed player.

The confidence heuristic applies to interactions in which there is no conflict of interest and the players are therefore motivated solely to coordinate their actions for mutual benefit. It clearly cannot apply to strategic interactions in general. For example, a used-car dealer may confidently assure a buyer about the quality of a car being sold, but this is likely to be treated with scepticism by the buyer, even though the buyer knows that the dealer has superior knowledge of the car’s quality. In strategic interactions in which players can benefit selfishly by misleading each other, the confidence heuristic is evidently inapplicable. However, a question that arises naturally from theoretical and empirical research on coordination games is as follows. What happens in games of incomplete and asymmetric information in which communication is possible, but deceptive signalling is potentially profitable to the better-informed players? Do these players actually use deceptive confidence signalling, in particular response latency manipulation of accurate information, to gain a selfish advantage? Recent research has highlighted instances in which even telling the truth can lead to deception, when players tell the truth with the expectation of being mistrusted^11^ or send true messages with the expectation that their co-player/receiver will not follow these true messages^12^. We investigate whether lying or telling the truth is sometimes accompanied with deceptive confidence signalling as a way to deceive.

In cooperative interactions, the assumption that communication is honest is reasonable, whereas in mixed-motive interactions it is undermined, according to interpersonal deception theory^13,14^, by participants’ attempts to conceal their uncertainties^15^, and in such interactions players may engage in strategic misrepresentation of their confidence. As a consequence, competitive strategic interactions tend to foster overconfident signalling^16,17^ to influence others and thereby benefit themselves^18^, frequently using nonverbal confidence signalling because of its plausible deniability^19^. For the same reason, people sometimes withhold information to underplay their own knowledge for strategic reasons^19,20^.

The research described below examines whether strategic deception is used in interactions involving players with conflicting interests, and if so, whether this is implemented with confidence signalling, perhaps through withholding information from the other participant at the start of the interaction. This is a new research question with a new research paradigm for implementing games of incomplete information experimentally. We therefore also investigated individual differences that might be related to strategic deception in such circumstances. Apart from a general measure of personality (Big 5) we specifically measured prosociality (SVO), because recent research has shown that prosocial people show less “knowledge hiding” in workplace settings^21^, and send fewer deceptive messages^22^. We also added the Dark Triad personality measure because research has shown that these traits are associated with knowledge hiding and deceptive behaviours in the workplace^23,24^.

## Methods

**Fig. 1:**
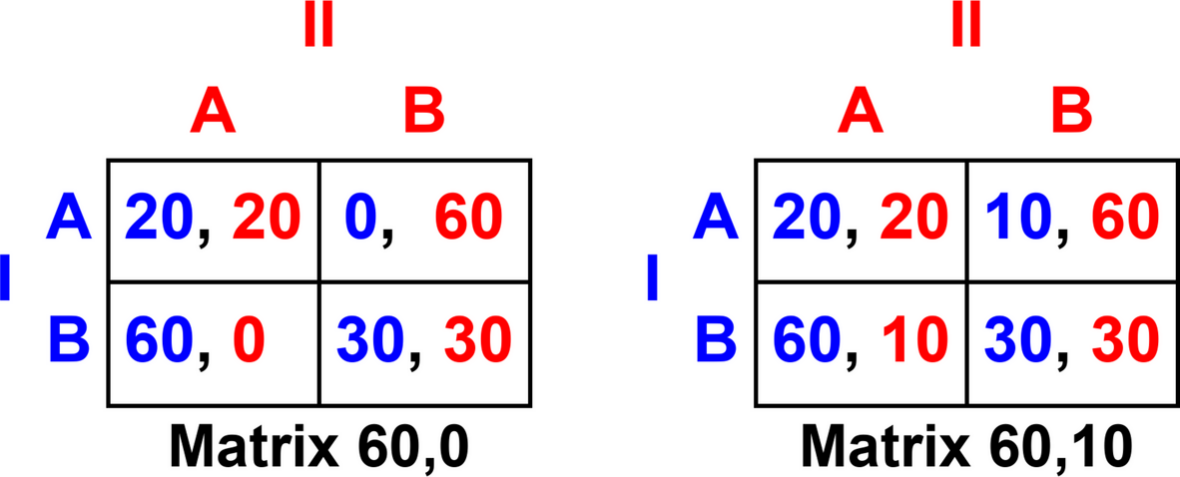
Deadlock game: payoff matrices for two versions used in the study. In each payoff matrix, Player I chooses between the rows, Player II chooses between the columns, and the payoffs to Players I and II are shown in that order in the cell where the two choices intersect. The Deadlock game is defined by the ordinal relations of the payoffs, which are identical in the two versions. The only difference is that the lowest payoff for both players is 0 in Matrix 60,0 and 10 in Matrix 60,10.

To investigate these problems, we incentivized participants with two versions of the mixed-motive Deadlock game shown in Fig. 1. The participants were not shown these payoff matrices, but in every experimental trial, one of the matrices was used to determine the incentive scheme, which was explained verbally to the participants. Pairs of participants in our study had the task of deciding, in a series of diagrams presented to them, which of two shapes was closest in size to a target shape. Participants were told that for each trial the payoff was 30 each if they both chose the right shape; 20 each if they both chose the wrong shape; 60 to a participant who alone chose the right shape; and either 0 or 10 (on different trials) to a participant who alone chose the wrong shape. Those outcomes correspond respectively to Player I’s payoffs in the following cells in Fig. 1: (B, B); (A, A); (B, A) or (A, B). Participants were told that these payoffs represented pounds sterling that they could earn if they were one of two winners of a lottery following the conclusion of the study (explained below).

Like the well-known Prisoner’s Dilemma game that it resembles, the Deadlock game, Game 8, p. 25 in a standard taxonomy of games^25^, has a unique Nash equilibrium at (B, B) – equivalent to (D, D) in the more conventional labelling of the Prisoner’s Dilemma game – and in both games B is a dominant strategy for both players. But in the Deadlock game, unlike the Prisoner’s Dilemma game, the payoffs at (A, A) are worse for both players than the payoffs at (B, B), and neither player has any temptation to play A under conditions of complete information. However, if one of the players is less well informed about which combinations of choices lead to which payoffs, the player with stronger evidence can gain the maximal payoff of 60 by secretly choosing B and tricking the other player, who does not have complete information of the payoffs, into choosing A. The Deadlock game has therefore been used to model one-sided bargaining offers under conditions of incomplete information, as when a seller knows the quality of a product, such as the used car in the example given earlier, but the buyer does not^26,27^.

In both versions of the game in Fig. 1, players do better by jointly agreeing to choose B (the right answer in all the problems in our study) than by jointly choosing A (the wrong answer), but an unscrupulous player with superior information can gain the maximum payoff and reduce the other player’s payoff to the minimum *sucker’s payoff* by choosing B while simultaneously persuading the other player to choose A. Our reason for including two versions of the game (one with 0 and the other with 10 for the sucker’s payoff) was that zero payoffs have special psychological significance in the “fast and frugal” heuristic, *Avoid the Worst*^28,29^, and also in regret theory^30,31^ and disappointment theory^32^. We manipulated this to discover whether people were more likely to exploit and deceive their co-player when they did not entirely rob them of all their winnings, although the presence or absence of zero sucker’s payoffs turned out to make no difference in our study.

### Participants

The participants were 110 undergraduate students (24 men and 86 women; mean age 19.59 years, *SD* = 1.94) recruited via an online self-booking system. We rewarded participants with course credits for their participation in the study and, using the matrices shown in Fig. 1, we incentivized them further with the *random lottery incentive system*, which has been shown to be valid^33^ and to elicit true preferences^34^. After testing all participants, we selected two randomly and rewarded them with the payoffs they received in one of their decisions, also chosen randomly; in the event, two participants earned £30 each.

A power analysis using G*Power^35^ for a 2 × 2 × 2 repeated-measures ANOVA with α = 0.05 and power 1 – β = 0.80 showed that a sample size of at least 30 is required to detect medium effect sizes of *f* = 0.25. For our multiple regression analysis, again with α = 0.05, power 1 – β = 0.80, and nine predictor variables, a sample size of at least 55 is required to detect medium effect sizes of *f*^2^ = 0.15. To provide a safe margin of error and the possibility of detecting smaller effects, we tested 110 participants after discarding data from three pairs of players who turned out to know each other – participants had been explicitly asked *not* to sign up to a testing session with a friend or acquaintance.

### Design

The experimental design was a 2 × 2 × 2 within-subjects experiment with three independent variables: Answer type (spoken/written), Evidence (weak/strong), and Matrix (60,0/60,10). For each trial, the accuracy of the choice made and the self-rated confidence were the primary dependent variables.

We used the following psychometric scales to measure individual differences among the participants: the Short Dark Triad Scale^36^ (SD3); the short-form Big Five Inventory–2^37^; and slider measure of social value orientation^38^ (SVO). The rationale for including the SD3 scale was to investigate possible influences of Narcissism, Machiavellianism, and Psychopathy on manipulative strategic deception in our experimental task. We included the Big Five Inventory–2 speculatively, to discover whether any of the major factors of personality (Extraversion, Agreeableness, Conscientiousness, Neuroticism, Openness) might possibly be associated with strategic deception. Finally, we included the SVO scale because it measures prosociality in strategic interactions and was therefore an obvious measure to include, given that our tasks were strategic interactions, and deceptive selfishness is the antithesis of prosociality.

### Materials

Each participant was given a different folder, containing 24 test trials (12 shape diagrams, each presented twice: once for Matrix 60,0 and once for Matrix 60,10). Matrix order and trial order (1 to 12, or 12 to 1) were counterbalanced to control for possible order effects. The A and B labels in the matrices in Fig. 1 do not necessarily match the A and B labelling in the tasks used and dataset, because we randomised which answer was correct for the strong-evidence player. Each participant was given strong evidence on six randomly selected trials and weak evidence on the remaining six for each matrix. Participants also received booklets with the experimental instructions and response sheets.

**Fig. 2:**
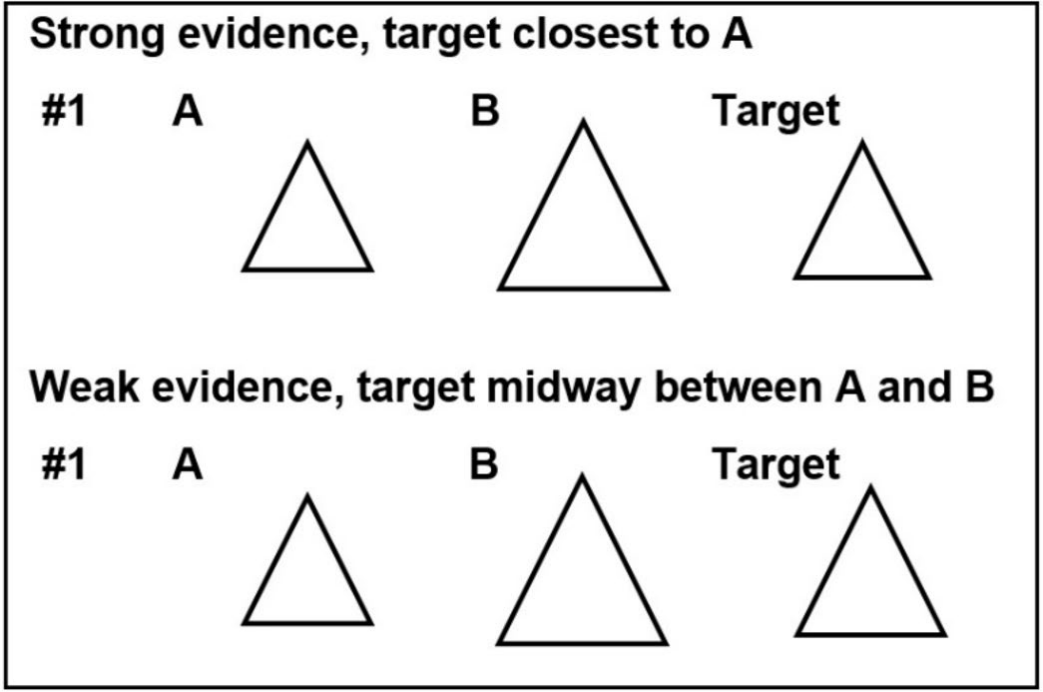
One of the test stimuli, showing versions for the players with strong and weak evidence. To control for labelling effects, on some trials the correct answer was A and on others B, although in Fig. 1, the correct answer is B in both matrices.

The test shapes used in our study were selected from materials already validated by Pulford et al. (2018, Study 2)^9^. Test stimuli from a typical trial are shown in Fig. 2.

On each trial, both participants saw the same two test shapes A and B but different target shapes: for one participant, one of the test shapes was close in size to the target shape (strong evidence); for the other participant, the two test shapes were equally distant in size from the target shape (weak evidence). The instruction booklet made participants aware of the informational asymmetry by stating that the two participants may “have different quality information”. Participants were not informed who had the better quality (strong) evidence on each trial, and the position of the six strong-evidence materials in the block of 12 trials was randomized when the materials were prepared. Participants played two blocks of 12 trials each. After the size judgment task, they completed an online questionnaire including the psychometric scales mentioned above.

### Procedure

Participants were tested one pair at a time. The pair members sat on opposite sides of a table, divided by a low partition that obscured their view of each other’s test materials and answer sheets but allowed face-to-face communication. After giving informed consent, participants recorded their ages and genders on their answer sheets and read the instructions outlining the goal of the task and the payoffs. The experimenter answered any questions and reminded them of the payoffs and incentive scheme.

On each trial, participants were given up to two minutes to examine the diagram, discuss which shape, A or B, was closest in size to the target, and write down their individual answers. All sessions were audio recorded. In the testing sessions, the experimenter observed the participants and manually recorded, for each trial, who was the first to suggest an answer (and whether it was A or B), and the last spoken answer, stated or implied, of each participant. Then, for each trial, participants wrote down their personal final answers (A or B), what they thought the other player chose (A or B), how confident they were about their answers, on a scale from 0 (*not at all sure that I chose the right shape*) to 100 (*completely sure that I chose the right shape*), and how likely they thought it was that they had been deceived by the other player, on a scale from 1 (*not at all likely*) to 7 (*highly likely*).

No feedback regarding the accuracy of answers was given, potentially enabling deception to go unnoticed, or at least unconfirmed. After completing the first block of 12 trials, participants were given instructions for the second block. After completing both blocks, participants completed the three personality questionnaires.

### Data analysis

Participant pair 15 settled on a high-level strategy in which it was clear from their spoken comments and written responses that they systematically “agreed to disagree” on every trial. Data from this pair were removed from our data analysis. No other data were removed from the dataset. Participants very occasionally omitted to answer every question in responding to the psychometric scales, and in such cases scale scores were calculated from the means of the answered items.

Accuracy was operationalized as the correspondence between the answer given and the answer implied by the strong evidence, taking the value of 1 if the two answers coincided, and 0 if they did not coincide. We computed the percentage accuracy when participants had strong evidence and when they had weak evidence, for each matrix (over six trials) and across both matrices (over 12 trials). We did this for both written accuracy and spoken accuracy (the last spoken answer, stated or implied). We used the accuracy values (0/1) rather than the payoff values to compare the two matrices, because the range of payoff values differed between the two matrices (0 to 60 and 10 to 60).

## Results

### Answer accuracy

**Fig. 3:**
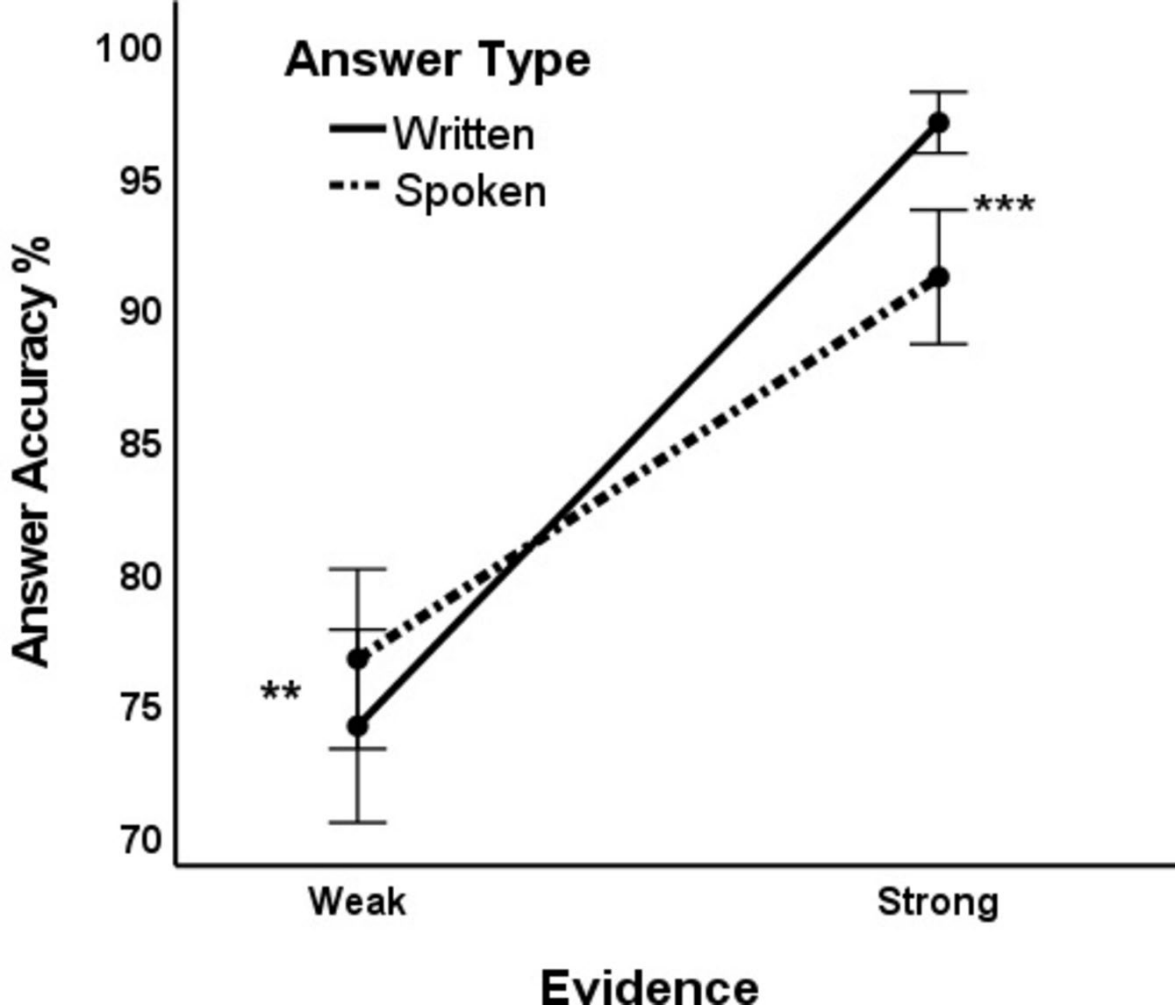
Interaction between answer type and evidence strength on answer accuracy. *** *p* < .001; ** *p* < .05. Error bars show 95% confidence intervals.

The percentage accuracy was entered as the dependent variable in a 2 (Answer type: spoken/written) × 2 (Evidence: weak/strong) × 2 (Matrix: 60,0/60,10) within-subjects ANOVA. We found a significant small to medium effect of answer type (spoken/written) on answer accuracy: *F*(1, 107) = 4.93, *p* = .03, partial η^2^ = 0.04, and a significant large effect of evidence (strong/weak): *F*(1, 107) = 111.24, *p* < .001, partial η^2^ = 0.51. There was no significant effect of matrix or interaction with it. Figure 3 shows the significant medium-sized interaction between answer type (spoken/written) and evidence (strong/weak) on answer accuracy: *F*(1, 107) = 18.66, *p* < .001, partial η^2^ = 0.15. Paired *t* tests showed that for participants with weak evidence, written accuracy was significantly lower than spoken accuracy (*M =* 74.07%, *SD* = 19.21 and *M =* 76.62%, *SD* = 17.89, *t*(107) = − 2.26, *p* = .03, *d* = − 0.22). This indicates the effectiveness of deception: the reason why less accurate written answers were given by players with weak evidence is that some of them were deceived into writing the wrong answer. However, for participants with strong evidence, written accuracy was significantly higher than spoken accuracy (*M =* 96.99%, *SD* = 6.08 and *M =* 91.13%, *SD* = 13.35, *t*(107) = 4.44, *p* < .001, *d* = 0.43), because a large minority of strong-evidence spoken responses were deceptive.

### Mean self-rated confidence

We ran a 2 × 2 repeated-measures ANOVA to test whether evidence (strong/weak) and matrix (60,0/60,10) affected participants’ self-rated confidence. Participants reported much higher confidence in their chosen answers after receiving strong evidence (*M* = 86.81, *SD* = 9.39) than weak evidence (*M* = 73.04, *SD* = 13.80), *F*(1, 106) = 191.79, *p* < .001, partial η^2^ = 0.64, thus suggesting that evidence strength had a significant and large effect on confidence. Matrix (60,0/60,10) had no significant effect on participants’ confidence: *F*(1, 106) = 0.01, *p* = .94, partial η^2^ < 0.001, and there was no significant interaction of Evidence × Matrix (*p* > .05).

### First spoken answers

The rest of the results relate to spoken behaviour (first spoken and final spoken) and written answers analysed by trials rather than by individuals. Previous literature^9,10^ indicates that being the first to suggest an answer is an important confidence signal, hence we report this first. On average, players with strong evidence were the first to suggest an answer on 75.54% (*SD* = 18.63) of trials, compared to 24.23% of trials (*SD* = 18.50) when they had weak evidence, *t*(107) = 22.41, *p* < .001, *d* = 2.16 (large). The correlation between how frequently they were the first to suggest an answer on the 12 trials when they had strong evidence versus the 12 trials when they had weak evidence was non-significant (*r* = .18, *p* = .06), indicating that being the first to suggest was primarily driven by situational factors, such as evidence strength, rather than individual differences, such as personality.

**Fig. 4:**
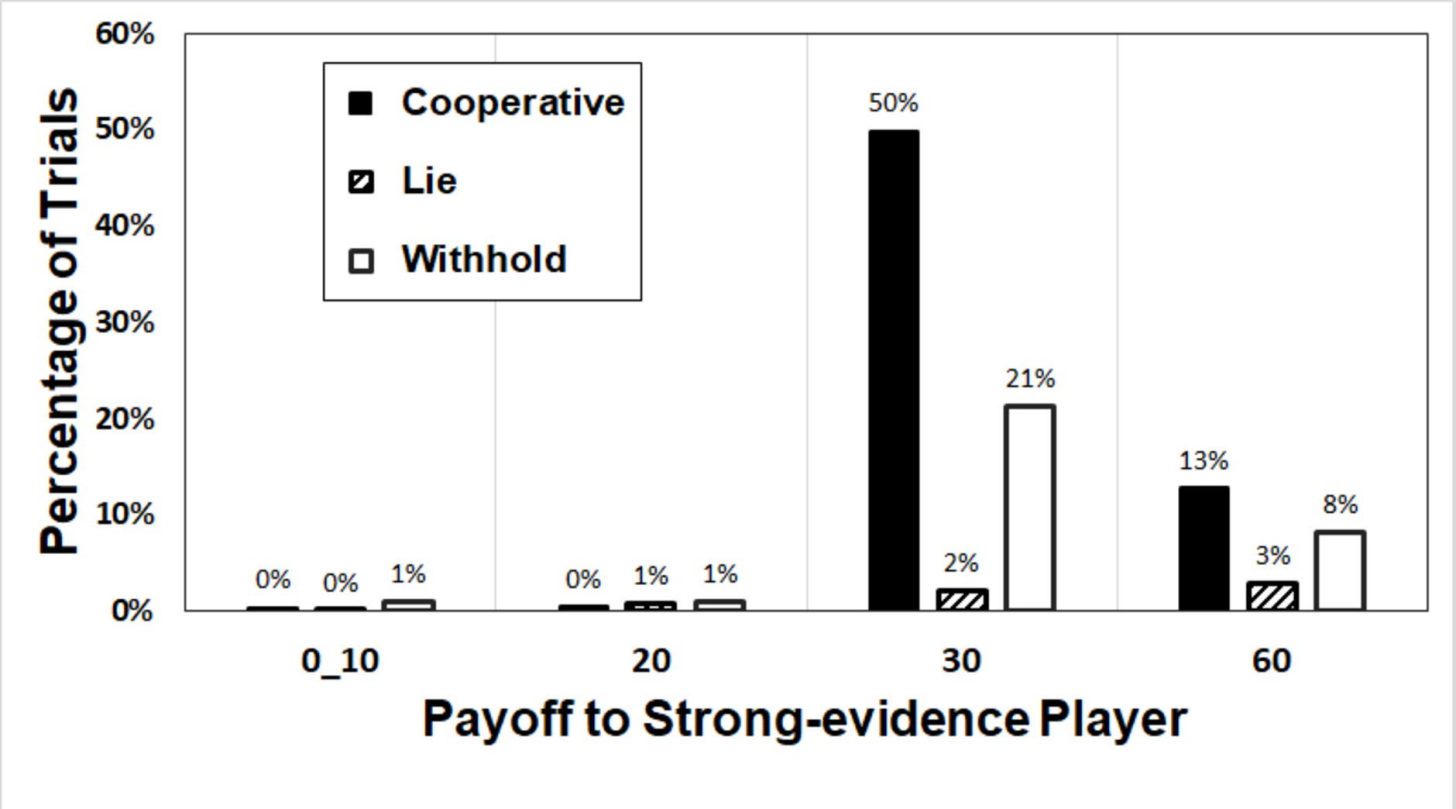
Percentages of trials on which strong-evidence players received particular payoffs after initially cooperating, lying, or withholding information.

Figure 4 shows the payoffs to the strong-evidence players, who presumably knew the right answer but could choose to lie about it to deceive the other player with the aim of securing a maximal payoff of 60. The payoffs to the strong-evidence players are broken down according to whether those players cooperated by suggesting the right answer to the weak-evidence player first, lied by suggesting the wrong answer first, or withheld the right answer at the start of the discussion at least until after the other player suggested an answer. Only 3% of these trials led to the strong-evidence players receiving one of the lowest two payoffs, shown in the left-hand half of the figure, because the right answer was, by definition, easy to identify on these trials. This serves as a manipulation check, confirming that the materials did in fact make it very easy for the strong-evidence players to identify the correct answers. Given that on 97% of trials the strong-evidence participant chose the right answer, the payoffs of 30 or 60 resulted from the answers given by the other (weak-evidence) players, as explained below.

**Fig. 5:**
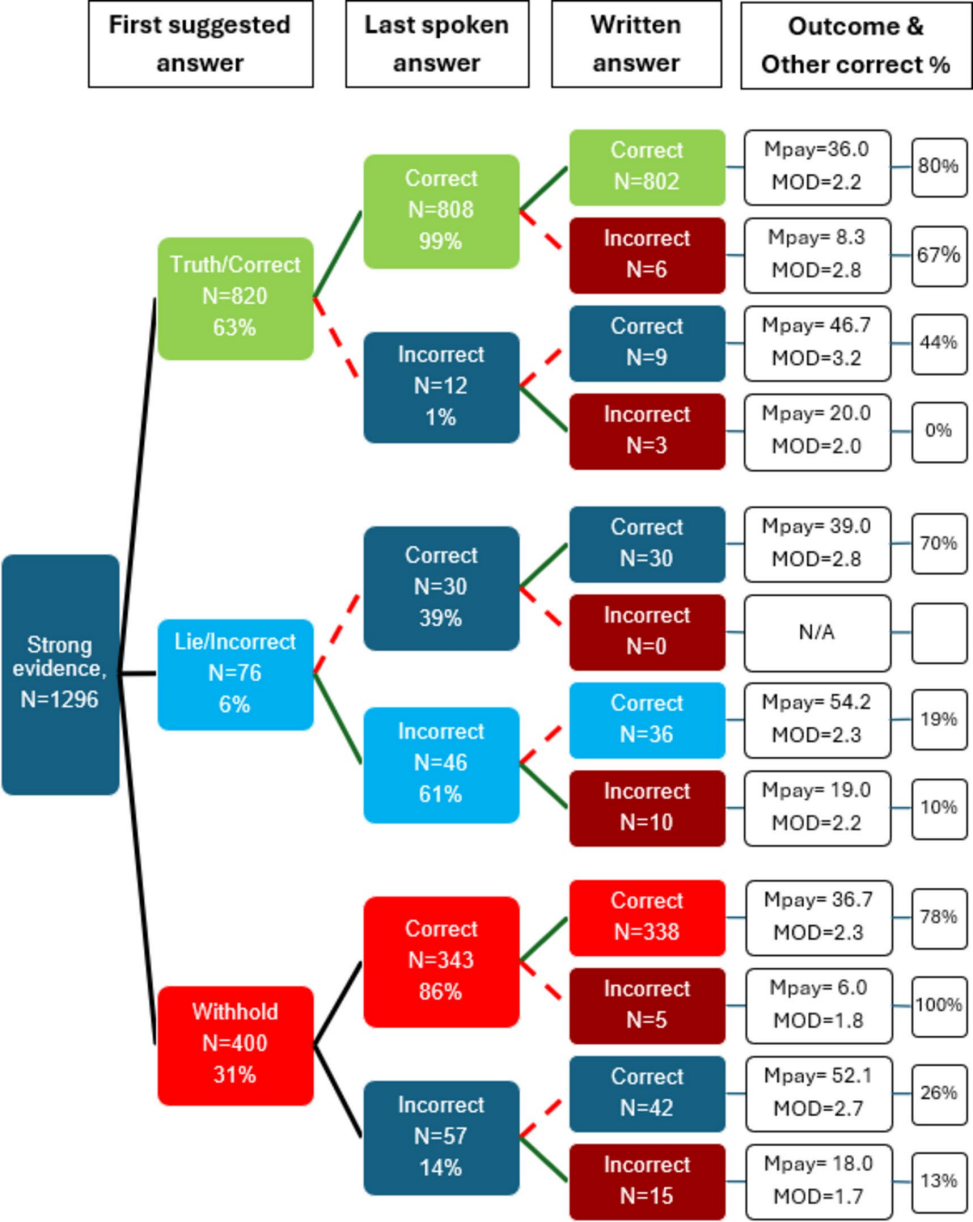
Initial communications of strong-evidence players and ensuing event paths. Colours represent different patterns of behaviour, the most common being as follows: The green path represents truthful/correct answers at all stages; the light blue path represents the verbal lies followed by written correct answer; and the red path represents initial withholding but then finally stating the correct answer verbally and in writing. Dashed lines indicate players’ changes of answer, continuous lines indicate consistency, or at least non-inconsistency. Mpay = mean payoff (out of 60); MOD = Mean perception of deception by the other player, on 1 to 7 (low to high) scale. The final column is the percentage of other (weak-evidence) players who picked the correct answer. All percentages are breakdowns of immediately preceding boxes.

The strong-evidence players initially withheld the right answer on a total of 31% of trials (white bars), and this led to them receiving the maximal payoff of 60 on 8% of all trials. These maximal payoffs must have resulted from the weak-evidence players choosing the wrong answers. On 21% of the trials, a mutually beneficial (30, 30) outcome resulted eventually, because on 86% of occasions the strong-evidence player relented and suggested the right answer after initially withholding it (see Fig. 5), or perhaps because the other player could determine the correct answer from other cues in the conversation.

The striped bars in Fig. 4 show that, on 6% of trials, the strong-evidence player was the first to suggest an answer and blatantly lied by suggesting the wrong answer. This resulted in the maximal payoff of 60 on only 3% of trials, where the lie was effective in determining the weak-evidence player’s answer, and a payoff of 30 on 2% of trials, in which the lie was either retracted or disregarded by the weak-evidence player.

The black bars in Fig. 4 show that the strong-evidence player was first to suggest an answer that was the right answer on 63% of trials. This is evidence of honest, cooperative strategic behaviour and, according to the confidence heuristic literature reviewed above, it confirms that answering first is a persuasive way to communicate that one has strong evidence. Remarkably, however, on 13% of trials (20.6% of all the initially cooperative trials) these honestly proffered first answers were not accepted by the weak-evidence players, who eventually chose the wrong answers, resulting in the highest payoff of 60 for the strong-evidence players and the lowest (sucker’s) payoff for the weak-evidence players. To understand these finding better, we plotted the course of communicative events in Fig. 5.

### Analysis of first and last spoken and written answers

Figure 5 depicts all trials across both matrices: the behaviour of the strong-evidence player across all 1,296 trials, and what followed. We chose not to report matrices separately, because three *t* tests showed no significant difference in the strategies occurrence split by matrix (all *p* > .05). Following the top path first, we find that on 63% of trials, the strong-evidence player gave the correct answer as the first suggested answer, on 99% of those trials repeated this suggestion as a last spoken answer, and on all but six of those trials (99%) recorded the same suggestion as a personal written answer after the discussion. In the right-hand column we see that on 80% of those trials, the weak-evidence player trusted this information and also chose the correct answer. However, this means that on 20% of trials the weak-evidence player mistrusted this information and chose the incorrect answer, causing the mean payoff to the honest strong-evidence player to be slightly higher (36.0) than it would have been if the honest suggestion had invariably been accepted, in which case it would have been 30.0. On fewer than 1% of trials, the strong-evidence players used the strategy of being truthful at first and then switching to suggesting the wrong answer.

Looking at the middle row of the first column in Fig. 5, on 6% of trials the strong-evidence player was the first to suggest an answer that was a lie (suggesting the wrong shape). This lie was maintained as the last spoken answer on 61% of those trials, but then the strong-evidence player recorded the correct personal answer in writing and received the highest mean payoff of any strategy (54.2). On 19% of those trials the weak-evidence players must have suspected the deception, because they chose the correct answer.

From the bottom paths in Fig. 5, we see that the strong-evidence player initially withheld the right answer – was not the first person to suggest an answer – on 31% of trials. On 86% of those trials, the strong-evidence player relented and suggested the correct answer as a final spoken answer, although in a few (5) trials the strong-evidence player recorded the incorrect answer as a personal written answer. This strong-evidence strategy of withholding and then being truthful led to 78% of the weak-evidence players believing this final spoken answer and choosing the right answer themselves, and the strong-evidence players gained a mean payoff of 36.7, very close to the mean payoff of the fully cooperative truth-telling strong-evidence players. The mean perception of deception by the weak-evidence players was low and similar in both situations, hence 78–80% of weak-evidence players also ended up with the correct answer as a result of these interactions. The remaining 20–22% of weak-evidence players who ended up with the wrong answer may have interpreted their co-player’s truthful answer as a deception attempt or, strong-evidence players may have shared the correct answer with the expectation of being mistrusted.

In 42 instances (3.2% of all trials), strong-evidence players initially withheld the right answer but then suggested the wrong answer, then finally wrote down the correct personal answer and ended up with a very high average payoff of 52.1. This was a successful strategy, because only 26% of weak-evidence players then chose the correct answer, 74% of them being successfully misled. This combination of initially withholding and then lying triggered relatively low perceptions of deception by the weak-evidence players and was the second-most successful deceptive strategy by strong-evidence players for achieving the highest payoff, coming a close second to the consistent lying strategy.

In the “Written answer” column are a handful of instances (39 out of 1,296: 3% of answers) in which strong-evidence players chose and wrote down incorrect answers despite presumably knowing the correct answers. The payoffs are low in these instances, at best consistent with both players choosing the incorrect answer. This behaviour is not necessarily as incomprehensible as it appears, and we shall return to it in the Discussion.

### Individual differences

We used multiple regression to examine individual-difference variables that might predict the behaviour of strong-evidence players in the 12 trials on which they had the opportunity, at the start of the conversation, either to behave cooperatively by suggesting the right answer or to behave deceptively by suggesting the wrong answer or withholding the right answer. Table 1 shows that the total number of times strong-evidence players cooperated was significantly and positively predicted by their SVO Prosociality (cooperativeness and altruism) scores and by their Big Five factor scores for Conscientiousness and Neuroticism. Correspondingly, these three variables negatively predicted how many times the strong-evidence players withheld the correct answer until the other player had suggested an answer. It is worth noting that despite including measures of the dark triad of aversive and antisocial traits, we found no individual-difference variable that significantly predicted how frequently strong-evidence players blatantly spoke first with a lie.

**Table 1:** Predictors of initial communications by strong-evidence players: multiple regression statistics and standardized beta values.

Predictors	Cooperate	Lie	Withhold
Constant	1.49	0.87	9.64
Adjusted R^2^	0.22	− 0.01	0.15
*F*	4.26**	0.92	3.03*
SVO angle	0.33**	–0.19	–0.28*
SDT psychopathy	–0.11	0.04	0.10
SDT narcissism	–0.01	0.00	0.01
SDT machiavellianism	0.00	0.07	–0.03
BFI2 extraversion	–0.02	0.12	–0.04
BFI2 agreeableness	–0.17	0.08	0.15
BFI2 conscientiousness	0.42**	–0.18	–0.38**
BFI2 neuroticism	0.33**	–0.10	–0.31*
BFI2 openness	0.01	–0.01	0.00

*p < .01; **p < .001; *N* = 107.

## Discussion

The primary questions that motivated this research are answered by the results of our investigation. We have reported ample evidence that, in games of incomplete and asymmetric information in which players with superior information could use deceptive confidence signalling to gain a selfish advantage, such behaviour was indeed observed in a significant proportion – roughly one-third – of trials. Furthermore, our results confirm that the most common and effective mechanism through which such deception was implemented was by players with superior information withholding the right answer from their less-informed co-players, at least until after their co-players had suggested their own answers. This may have been an attempt to appear less confident in the answer, and it seems to have been effective, because on 8% of all trials the maximal payoff of 60 was achieved via this withholding strategy. Indeed, in attempting to obtain the maximal payoff of 60, the most effective strategy after withholding was to give the wrong answer. This was not a frequently used strategy, but when it was used it did result in high payoffs, almost as high as those achieved by lying consistently. These findings are in line with previous findings on withholding of information^21–24^.

Communicating the wrong answer was clearly an intentional act because the tasks we presented were not difficult for the person with strong evidence, as evidenced by their 97% written accuracy rate. Our research shows that true messages were often given after initially withholding the correct answer until after the other person had suggested an answer. Withholding then telling the correct answer was a frequent strategy (343 out of 1296 trials: 26.5%). If the situation had been presented as a cooperative one, then there would have been no reason to withhold information. The asymmetrical information situation presented an opportunity to get higher payoffs and this was achieved in part by deceptive confidence signalling, in particular response latency manipulation of accurate information, to gain a selfish advantage. Telling the truth alongside deceptive confidence signalling is an appealing way to deceive because appearing low in confidence reduces the probability that people will believe the message that you are sending, due to the confidence heuristic, but it is not outright lying.

Lying, whether outright or after an initial attempt at withholding, yielded the most money. Outright lying from the start of the conversation was relatively rare – it was attempted on only 6% of trials – but was effective when it was tried, and the lie was maintained. The relative infrequency of outright lying is consistent with the findings of Alempaki et al.^39^, who reported that strategic deception is typically implemented through evasions – messages that withhold, bend, or distort the truth – rather than through direct lies, because evasions are less psychologically costly to the deceiver. This interpretation is consistent with *enthymematic parsimony* – arguments in which some elements are left unstated to optimize cognitive resource consumption^40^. Overall, our main findings corroborate those of Pulford et al.^9^ (2018) and show that the same confidence signalling strategy of being the first person to suggest an answer, which is used in strategic interactions in which people are motivated to coordinate their actions, is used quite frequently to deceive less well-informed co-players in interactions in which the individuals’ interests do not coincide. By withholding information and not suggesting an answer first, the impression of lower confidence is signalled to the co-player, who may then be more likely to stick with their own best guess, which is more likely to be incorrect because they have weaker evidence.

The fact that the accuracy level in the weak-evidence condition is much lower than in the strong-evidence condition indicates that the weak-evidence players were not immediately corrected by the strong-evidence players, which they would have been in the absence of any deceptive behaviour of the strong-evidence players. The lower accuracy in the weak-evidence condition shows that the strong-evidence players’ knowledge was not fully and openly shared and accepted by the weak-evidence players. The gap in accuracy between the final spoken answer and the written answer (5.86%) in the strong-evidence condition must be due to blatant lying. The gap in accuracy between the final spoken and written answers in the weak-evidence condition (–2.55%) must be due to the weak-evidence players changing their answers and rejecting the correct answer, presumably due to a lack of trust in the high-evidence players’ suggestions.

One of the more surprising findings of our study was that on 13% of trials, the weak-evidence players rejected the honest and cooperative suggestions of the strong-evidence players and insisted on choosing the wrong answers, with the result that they earned the sucker’s payoff for themselves, as shown in Fig. 4. This behaviour seems to fly in the face of truth-default theory^41^, according to which people generally default to believing others. Previous research^48,49^ has suggested that people tend by default to accept communicated information, at least provisionally. However, when their interests conflict with those of the communicator, our findings suggests that they sometimes reject the communicated information immediately, in accordance with the notion of *epistemic vigilance*^50,51^. Although this cynical and self-defeating type of response was relatively rare, it was sufficiently common to demand an explanation, and a clue may be provided by similarly cynical and self-defeating anti-vaccine beliefs of a small but significant proportion of people in population surveys. Hornsey et al.’s (2018)^42^ large-scale, exhaustive, and highly cited research into the psychological roots of such beliefs suggests that they are characteristic of people who score high on measures of conspiratorial thinking (not relevant to our current research) and *reactance*^42^, reactance being a tendency to respond to attempted persuasion as a threat to freedom of choice, eliciting a motivated rejection of what is being suggested. In future research, it would be useful to measure reactance proneness directly, with a scale such as the Hong Psychological Reactance Scale^43^ (HPRS), to check whether it does indeed explain self-defeating responses of the type observed in our study. Truth-default theory may be relevant to many social interactions but can hardly apply uniformly to strategic interactions in which there are conflicts of interest.

An aspect of the strong-evidence players’ choices is also difficult to understand, at least initially. It is apparent in Fig. 5, in the column headed “Written answer” that, on a small number of trials, strong-evidence players wrote down incorrect answers in spite of presumably knowing the correct answers and having high confidence. On these trials, they received low payoffs, or at best payoffs of 20 resulting from both players choosing the wrong answer. This anomalous type of responding may be random error or noise, or misinterpreting the other player’s intentions, or could be explained, in large part at least, by *inequality aversion*, a phenomenon that is well understood at a theoretical level and is frequently observed in experimental games^44–46^. Strong-evidence players who for whatever reason felt that they had not persuaded their weak-evidence partners of the correct answer during their discussions may have chosen to record incorrect written answers themselves to ensure that both players received equal payoffs. This implies that they could have exploited the weak-evidence players by choosing the correct answers, but if they did so, then the weak-evidence players would have received the lowest sucker’s payoff of either 0 or 10 depending on which matrix was being used. To some strong-evidence players, the prospect of earning 60 while their partners receive derisory payoffs may have seemed repugnant, and is consistent with the concept of a stable prosocial phenotype^47^.

The results reported above also include findings about individual differences in strategic deception. Deceptive withholding behaviour was quite strongly predicted by individual differences of SVO Prosociality (cooperativeness and altruism), in line with previous findings on knowledge withholding^21,22^, and also predicted by the Big Five personality factors of Conscientiousness and Neuroticism. People who scored high on these three traits, when they were the strong-evidence player, were significantly more likely to initiate negotiations with their weak-evidence co-players cooperatively and honestly, by revealing the right answer (which was obvious to them and about which they felt confident) and less likely to withhold. It is obvious why SVO Prosociality was strongly predictive of cooperative and honest negotiation, because it is precisely a measure of cooperativeness and altruism in strategic interaction. It is not difficult to understand why the Big Five personality factor of Conscientiousness also strongly predicted cooperative and honest negotiation and negatively predicted initial withholding. Among its most salient component traits is reliability, and strategic deception is quintessentially unreliable. Why the Big Five personality factor of Neuroticism also predicted cooperative honesty is harder to explain. It includes component traits of nervousness and tenseness, and low Neuroticism equates to emotional stability. In our study, strong-evidence players who scored high on Neuroticism may have felt nervous about being caught out in a lie, or anxious about feeling guilt and remorse following a lie. In either case, to those among them who might otherwise have been tempted to lie, cooperative honesty may have seemed a safer option, and withholding may have appeared less appealing.

Our research adds to the recent research on omission lies, such as by Leal et al.^52,53^, when lie tellers are truthful but omit information. While our research sheds light on confidence in deceptive situations, the situation our participants were placed in was not high-stakes, and the lie to be told was not very cognitively demanding. In real-world interactions that require recall from memory and more cognitively challenging fabrication of information, being the first person to suggest an answer may be different. For one thing, reaction times will be longer in such situations^54^. There are many other potential confidence signals, such as loudness^10^ or the certainty level of the words used^55^ that may also matter in deceptive situations and are worthy of future investigation alongside our finding of strategic withholding of the correct information. Our study shows that it is not difficult to implement incomplete information in an experimental study, in addition to previously published techniques^2–7^, but this also suggests a possible limitation on the generalizability of our findings. Only future research using different techniques of creating incomplete information can establish the external validity of our study.

## Data Availability

All data underlying the findings are openly available at OSF, accessible via https://osf.io/c78y6/.
